# Ion Channels of Pituitary Gonadotrophs and Their Roles in Signaling and Secretion

**DOI:** 10.3389/fendo.2017.00126

**Published:** 2017-06-09

**Authors:** Stanko S. Stojilkovic, Ivana Bjelobaba, Hana Zemkova

**Affiliations:** ^1^Section on Cellular Signaling, Eunice Kennedy Shriver National Institute of Child Health and Human Development, National Institutes of Health, Bethesda, MD, United States; ^2^Institute for Biological Research “Siniša Stanković”, University of Belgrade, Belgrade, Serbia; ^3^Department of Cellular and Molecular Neuroendocrinology, Institute of Physiology Academy of Sciences of the Czech Republic, Prague, Czechia

**Keywords:** gonadotrophs, gonadotropin-releasing hormone, voltage-gated channels, ligand-gated channels, electrical activity, calcium signaling, luteinizing hormone secretion

## Abstract

Gonadotrophs are basophilic cells of the anterior pituitary gland specialized to secrete gonadotropins in response to elevation in intracellular calcium concentration. These cells fire action potentials (APs) spontaneously, coupled with voltage-gated calcium influx of insufficient amplitude to trigger gonadotropin release. The spontaneous excitability of gonadotrophs reflects the expression of voltage-gated sodium, calcium, potassium, non-selective cation-conducting, and chloride channels at their plasma membrane (PM). These cells also express the hyperpolarization-activated and cyclic nucleotide-gated cation channels at the PM, as well as GABA_A_, nicotinic, and purinergic P2X channels gated by γ-aminobutyric acid (GABA), acetylcholine (ACh), and ATP, respectively. Activation of these channels leads to initiation or amplification of the pacemaking activity, facilitation of calcium influx, and activation of the exocytic pathway. Gonadotrophs also express calcium-conducting channels at the endoplasmic reticulum membranes gated by inositol trisphosphate and intracellular calcium. These channels are activated potently by hypothalamic gonadotropin-releasing hormone (GnRH) and less potently by several paracrine calcium-mobilizing agonists, including pituitary adenylate cyclase-activating peptides, endothelins, ACh, vasopressin, and oxytocin. Activation of these channels causes oscillatory calcium release and a rapid gonadotropin release, accompanied with a shift from tonic firing of single APs to periodic bursting type of electrical activity, which accounts for a sustained calcium signaling and gonadotropin secretion. This review summarizes our current understanding of ion channels as signaling molecules in gonadotrophs, the role of GnRH and paracrine agonists in their gating, and the cross talk among channels.

## Introduction

Gonadotrophs are the anterior pituitary cell lineage specialized for synthesis and release of two gonadotropins, such as follicle-stimulating hormone and luteinizing hormone (LH) (1). In addition to genes encoding beta subunits of gonadotropins, *Fshb* and *Lhb* (2, 3), gonadotrophs are defined by at least two other genes not expressed in other secretory pituitary cell types, such as gonadotropin-releasing hormone (GnRH) receptor (GnRHR) gene (*Gnrhr*) (4) and dentin matrix protein 1 gene (5). Together with thyrotrophs, gonadotrophs express the *Cga* gene encoding the α glycoprotein subunit (6). Ontogenetically, the lineage commitment is associated with the expression of the orphan nuclear receptor NR5A1, a transcriptional factor that also plays a role in the expression of gonadotroph-specific genes in the postnatal animals (7).

Gonadotrophs are neuron-like; they express numerous voltage-gated sodium (Na_v_), calcium (Ca_v_), potassium (K_v_), and chloride channels at the plasma membrane (PM), and fire action potentials (APs) spontaneously (8). These cells also express ligand-gated ion channels at PM, which activation by hypothalamic and intrapituitary ligands leads to increase in firing frequency and facilitation of Ca^2+^ influx and hormone release (9). The function of gonadotrophs is regulated by several Ca^2+^-mobilizing receptors capable of modulating electrical activity and AP-dependent Ca^2+^ influx and hormone release (10). The main Ca^2+^-mobilizing receptor for these cells is GnRHR, signaling through heterotrimeric G_q/11_ proteins (11), which α subunit activates phospholipase C-β1, leading to generation of inositol-1,4,5-trisphosphate (IP_3_) and diacylglycerol (12) and release of Ca^2+^ from endoplasmic reticulum (ER) through IP_3_ receptor (IP_3_R) channels (9).

Here, we focus on the role of ion channels in electrical/Ca^2+^ signaling and Ca^2+^-controlled cellular functions in gonadotrophs. We will first review the expression and roles of voltage-gated channels in spontaneous excitability and accompanied Ca^2+^ influx in these cells, followed by description of additional channels contributing to facilitation or modulation of excitability of these cells. These include the hyperpolarization-activated and cyclic nucleotide-gated (HCN) channels, acetylcholine (ACh)-gated receptor (AChR) channels, γ-aminobutyric acid (GABA)-gated A-type receptor (GABA_A_R) channels, and ATP-gated receptor (P2XR) channels, all expressed at PM, and IP_3_R channels expressed at ER membranes.

## Signaling by Voltage-Gated Channels

The superfamily of voltage-gated ion channels of more than 140 members, including Na_v_, Ca_v_, K_v_, and numerous less selective channels, is one of the largest groups of signal transduction proteins (13). These channels are also expresses in gonadotrophs and account for spontaneous and receptor-controlled electrical and Ca^2+^ signaling (9).

**Figure 1:**
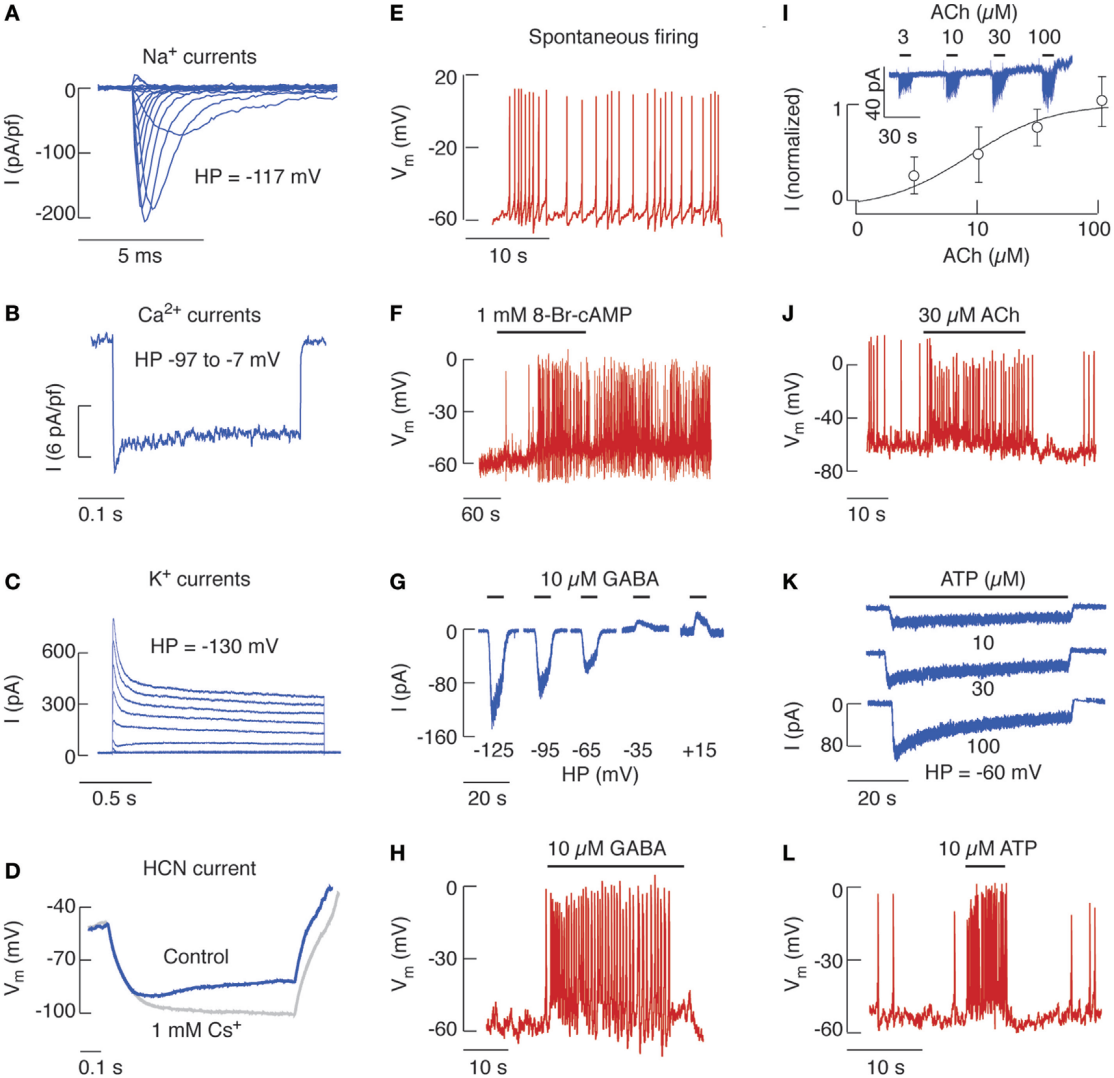
The expression of voltage-gated and ligand-gated ion channels in identified rat pituitary gonadotrophs. **(A)** Voltage-gated sodium current traces elicited by 100 ms voltage steps from holding potentials (HPs) of −117 mV. **(B)** Voltage-gated calcium current traces elicited by 400 ms voltage-steps. **(C)** Voltage-gated potassium current traces elicited by 1.5 s voltage steps. **(D)** Cells expressing HCN current display inward rectification in response to hyperpolarizing current pulses of −5 pA that are suppressed by 1 mM Cs^+^, a blocker of HCN channels. **(E)** Spontaneous electrical activity can be observed in about 50% of cultured gonadotrophs. **(F)** 8-Br-cAMP stimulates electrical activity. **(G)** γ-aminobutyric acid (GABA)-induced current recorded using gramicidin-perforated patch from cells held at different membrane potentials. **(H)** GABA-stimulated electrical activity. **(I)** Concentration-dependent effects of acetylcholine (ACh) on the amplitude of nicotinic current. (Top) Representative traces. (Bottom) Mean ± SEM values. **(J)** ACh-induced electrical activity. **(K)** Concentration-dependent effects of ATP on peak P2XR current response. **(L)** ATP-induced firing of action potentials. Derived from data shown in Ref. (16, 41, 50, 52, 63, 67); no permission is required from the copyright holder.

Nine members of Na_v_ channels are expressed in mammals, which contribute to the initiation and propagation of APs (14). The inward Na_v_ current has been identified in rat (15, 16), ovine (17), fish (18, 19) and mouse native (20, 21), and immortalized gonadotrophs (22, 23). Figure 1A shows traces of Na_v_ currents in cultured rat gonadotrophs. It appears that the level of Na_v_ channel expression is greater in these cells than in other secretory anterior pituitary types (16). Voltage-insensitive Na^+^ conductance is also present in all endocrine pituitary cells, including gonadotrophs (24, 25).

The Ca_v_ channels have a dual role in excitable cells: they generate inward currents that can initiate APs and are also critical for coupling of electrical signals on PM with physiological intracellular events by generating intracellular Ca^2+^ signals. There are 10 members of these channels that exhibit different electrophysiological and pharmacological properties (26). Pituitary gonadotrophs express at least inactivating T-type and non-inactivating L-type Ca_v_ currents, as documented in cultured cells from rat (16), mouse (21), ovine (27), fish (19), as well as in αT3-1 immortalized gonadotrophs (22). Figure 1B shows a representative trace of Ca_v_ current in rat gonadotrophs.

The K_v_ channels are composed of at least four functional classes: fast activating delayed rectifier, slow activating delayed rectifier (including M channels), A-type K_v_ channels, and ether-a-go-go-gene channels (28). Figure 1C illustrates total K_v_ currents in rat pituitary gonadotrophs, which are driven by several K_v_ channels. αT3-1 gonadotrophs (22) and native goldfish (19), rat (16) and ovine (29) gonadotrophs express delayed rectifiers, which expression is controlled by estrogens (29). The A-type K_v_ channels are also expressed in αT3-1 cells (22) as well as in native fish (18, 19, 30), frog (31), and rat (16, 32, 33) gonadotrophs. In rats, the expression level of these channels is much higher in gonadotrophs than somatotrophs (16). Functional M-type channels are expressed in mouse gonadotrophs and GnRH through a still uncharacterized signal cascade inhibits these channels (34). Moreover, our transcriptome study implies that a pulsatile GnRH application downregulates the expression of *Kcna2* (K_v_ 1.2) and *Kcnh6* (ether-a-go-go), while it upregulates *Kcnk10* (outward rectifier) and Na^+^/Ca^2+^ exchanger *Slc24a3*, indicating that GnRHR may indirectly be involved in regulation of cell excitability (5).

Calcium-activated K^+^ channels (K_Ca_) are composed of two families: three small-conductance K^+^ (SK) channels and one intermediate-conductance channel are members of the first family and the high-conductance K^+^ (BK) channels belong to the second family. These channels are activated by elevation in cytosolic Ca^2+^ and play a critical role in control of firing properties of excitable cells (35), including pituitary cells (36). The expression of SK channels is well documented in fish (37), rat (38, 39), mouse (40), and ovine gonadotrophs (17), and the level of their expression is dependent on estradiol (20). Whole-cell current recordings confirmed the presence of BK current in several pituitary cell types but not in gonadotrophs (16).

Gonadotrophs also express the hyperpolarization-activated and cyclic nucleotide-gated (HCN) channels (41), which are permeable to both K^+^ and Na^+^ and play a critical role in cardiac rhythmicity (42). As their name indicates, HCN channels are activated by voltage (Figure 1D) and cyclic nucleotides. Rat gonadotrophs and other pituitary cell types also express the cation-conducting transient receptor potential (TRP) cation-like channels (43), initially characterized by their role in *Drosophila* phototransduction (44). Mouse gonadotrophs express TRPC5 subtype of these channels, which are activated by GnRH and promote Ca^2+^ influx (45). Finally, Ca^2+^-activated non-selective cationic currents are present in rat gonadotrophs, but the nature of these channels has not been identified (46).

The expression of voltage-gated channels in gonadotrophs makes them electrically excitable cells, i.e., capable of exhibiting regenerative and propagated APs spontaneously or in response to stimulation. In general, the membrane potential (*V*_m_) of single gonadotrophs in culture is not stable but fluctuates from resting potentials of −60 to −50 mV due to spontaneous activity of hyperpolarizing and depolarizing channels. When the depolarization waves reach the threshold level, gonadotrophs fire tall and narrow APs (Figure 1E), with spiking frequency of ~0.7 Hz, amplitude of more than 60 mV, and half-width of about 50 ms (47). Ovine gonadotrophs also fire single APs spontaneously (17). In contrast to gonadotrophs, other pituitary cell types predominantly exhibit bursting pattern of spontaneous electrical activity, i.e., periodic depolarized potentials with superimposed small-amplitude spikes (47–50).

Depolarizing currents are pacemaking currents, accounting for a gradual reduction of PM resting potential toward the threshold for AP firing, and spike depolarization currents, accounting for the upstroke of an AP. The nature of channels contributing to pacemaking depolarization in gonadotrophs is not well characterized. The ongoing work is focused on the potential role of background Na^+^ (24) and TRP channels (43) in this process. The cell permeable cAMP analog 8-Br-cAMP initiates AP firing in quiescent gonadotrophs (Figure 1F) and increases the frequency of spikes in spontaneously firing cells (41), an action consistent with the expression of HCN channels (Figure 1D) and/or protein kinase A-mediated phosphorylation of some other channels in gonadotrophs (51).

The main function of Na_v_ channels is to depolarize cells and generate the upstroke of the AP, controlling the firing amplitude in excitable cell. In gonadotrophs, they act in conjunction with Ca_v_ channels to generate APs (17) or Ca_v_ channels are exclusively responsible for the spike depolarization (52). Simultaneous measurements of *V*_m_ and [Ca^2+^]_i_ showed that the bulk Ca^2+^ levels are low (50–100 nM) in spontaneously spiking gonadotrophs, in contrast to spontaneously bursting lactotrophs, somatotrophs and GH_3_B_6_ cells, which generate much higher (300–1,200 nM) and clearly oscillatory Ca^2+^ transients (48, 52, 53). In gonadotrophs, AP-driven Ca^2+^ influx is below the threshold needed to trigger exocytosis (52), whereas the bursting type of electrical activity in lactotrophs and somatotrophs accounts for high basal hormone secretion (48, 52). Because in intact tissue pituitary cell lineages are organized as complex networks (54–56), further studies are needed to characterize the excitatory and secretory patterns in pituitary cells with preserved tridimensional structure.

## Signaling by Ligand-Gated Receptor Channels

Ligand-gated receptors channels are activated by chemical signals (ligands) rather than to changes in the *V*_m_. These proteins are typically composed two different domains: a pore forming transmembrane domain and an extracellular domain containing the ligand binding site. There are three families of these channels: the Cys-loop family of channels activated by ACh, 5-HT, GABA, and glycine (57), glutamate-gated receptor-channels (58), and ATP-gated purinergic P2XR channels (59). Pituitary gonadotrophs express GABA_A_R, nicotinic AChR, and P2X2R channels (9).

γ-Aminobutyric acid is acting through GABA_A_R and GABA_C_R channels permeable to Cl^−^; in the central nervous system, GABA usually silences electrical activity and Ca^2+^ signaling (60). However, in gonadotrophs GABA and muscimol, a GABA_A_R agonist, increase intracellular Ca^2+^, suggesting that chloride-mediated depolarization activates Ca_v_ channels. Furthermore, the GABA_A_R channel reversal potential for chloride ions is positive to the baseline *V*_m_ (Figure 1G), and the activation of these channels results in depolarization of cells and initiation of AP firing (Figure 1H) and stimulation of *Fshb* and *Lhb* expression (61) and LH release (62). The lower expression of cation/chloride transporter KCC2 in rat pituitary cells probably accounts for the depolarizing nature of GABA_A_R channels in cultured gonadotrophs (63).

The binding of nicotine, ACh, or other ligands to AChR channels stimulates cation (Na^+^ and K^+^ and for some neuronal subtypes Ca^2+^ as well) influx through a channel and generally results in membrane depolarization. Seventeen subunits of nicotinic AChR have been identified and were shown to assemble into a variety of receptor subtypes (64, 65). We have shown recently the expression of β2, β1, α9, and α4 mRNAs in cultured rat pituitary cells and β2, α4, and α1 in immortalized LβT2 mouse gonadotrophs. We also showed the expression of β2 subunit protein in gonadotrophs (50). These cells express nicotinic AChR channels capable of generating an inward current (Figure 1I) and facilitating electrical activity (Figure 1J) and Ca^2+^ influx (not shown). We also found that GnRHR stimulation downregulates gene expression of both α4 and α9 subunits (5, 50), suggesting that the expression of nicotinic AChR in gonadotrophs *in vitro* compensates for the loss of GnRH stimulation.

ATP is not only an intracellular molecule but is also released by cells and acts as an extracellular ligand for P2XR family of channels, composed of three subunits, each composed of a large ectodomain, two transmembrane domains and the N- and C-terminus facing the cytoplasm (59). In intact gonadotrophs, ATP-induced extracellular Ca^2+^-dependent rise in cytosolic Ca^2+^ (66). In voltage-clamped cells, extracellular ATP-induced non-oscillatory current composed of rapidly depolarizing, slowly desensitizing, and rapidly deactivating phases, with the peak amplitudes and the rates of current desensitization determined by ATP concentration (Figure 1K). In current-clamped gonadotrophs, ATP induces a rapid depolarization that initiated firing of APs in quiescent cells, an increase in the frequency of firing in spontaneously active cells (Figure 1L), and a transient stimulation of LH release (67). The biophysical and pharmacological investigations suggested that gonadotrophs express the P2X2R subtype of these channels (67). Consistent with this conclusion, the full size and several splice forms of P2X2 subunit were identified in pituitary gland (68).

ATP is released by GnRH-secreting GT1 cells and cultured pituitary cells and metabolized by ectonucleotidase (69). Furthermore, GnRH increases ATP release in cultured pituitary cells (66). In accordance with these observations, it has been shown that ATP is co-secreted with GnRH from the median eminence into the hypophyseal-portal vasculature in ovariectomized sheep and that gonadotrophs have intrinsic ability to metabolize ATP in the extracellular space (70). This is consistent with the autocrine actions of extracellular ATP, where this molecule amplifies GnRH-induced Ca^2+^ signaling and LH secretion by activating P2X2Rs (67, 70). Pituitary cells other than gonadotrophs also express pannexin-1 and -2 channels (71), which contribute to ATP release in the extracellular medium in cultured pituitary cells (72). Thus, ATP and its degradation products ADP and adenosine may serve as paracrine factors to provide a cross talk between cell lineages within the pituitary gland *via* P2X2R (67), P2X4R (73), G-protein-coupled P2YRs (74), and adenosine receptors (75). By physical association with P2XRs, pannexin-1 may also provide a mechanism for autocrine control of functions of pituitary cell types expressing both proteins (76).

## Signaling by Channels Expressed in ER Membranes

Two families of structurally and functionally similar Ca^2+^ release channels, ryanodine receptors and IP_3_Rs, are expressed in the ER membrane. Ryanodine receptors account for intracellular transduction and translation of PM electrical signals by Ca^2+^-induced Ca^2+^ release from ER, whereas IP_3_Rs are activated by Ca^2+^-mobilizing receptors. In non-excitable cells, the IP_3_R-induced depletion of ER-Ca^2+^ stores facilitates Ca^2+^ influx through store-operated Ca^2+^-conducting PM channels. Two proteins, named stromal-interacting molecule and Orai, are critical for this Ca^2+^ entry pathway (77). IP3Rs are expressed in all secretory pituitary cells as indicated by ability of numerous Ca^2+^-mobilizing agents to trigger Ca^2+^ release from ER (9). In contrast, no conclusive evidence was presented about the expression and role of ryanodine receptors and Orai channels in gonadotrophs and other secretory pituitary cell types (36).

**Figure 2:**
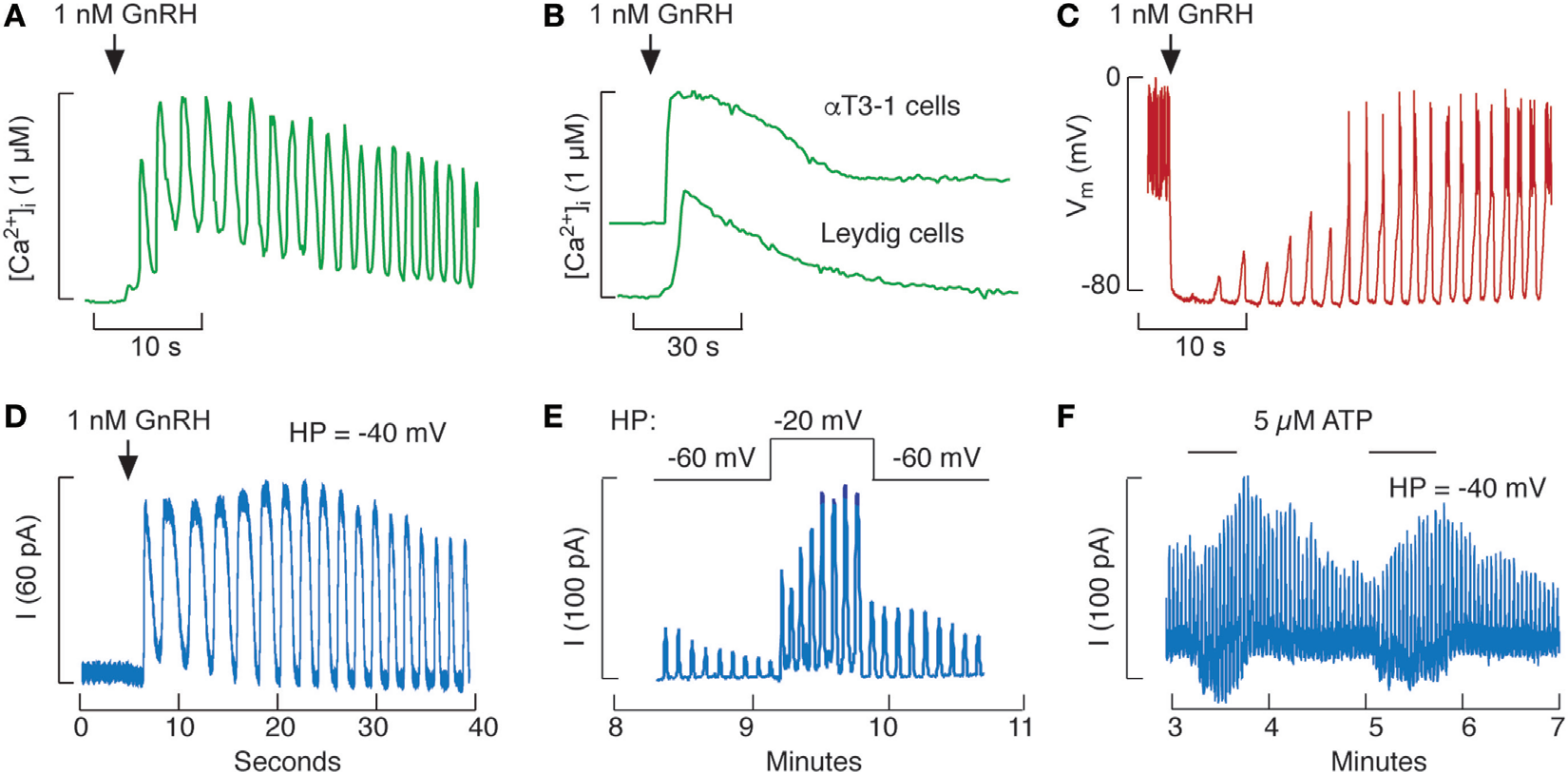
Influence of Ca^2+^ mobilization on excitability of pituitary gonadotrophs. **(A,B)** Gonadotropin-releasing hormone (GnRH)-induced calcium oscillations in rat pituitary gonadotrophs **(A)** and non-oscillatory calcium signals in immortalized αT3-1 pituitary gonadotrophs and testicular Leydig cells **(B)**. **(C,D)** GnRH-induced membrane potential **(C)** and small-conductance K^+^ (SK) current **(D)** oscillations in rat gonadotrophs. **(E,F)** Increase in the amplitude of GnRH-induced SK current oscillations by depolarization **(E)** and activation of P2X2R channels by extracellular ATP **(F)** in rat gonadotrophs. Current oscillations were initiated by 0.1 nM GnRH **(E)** and 1 nM GnRH **(F)**. Calcium recordings were done in intact Indo-1-loaded cells **(A,B)**, whereas electrophysiological recordings were done in nystatin-perforated cells voltage-clamped **(D–F)** or in current-clamped cells **(C)**. *V*_m_, membrane potential; I, SK current; HP, holding potential. Derived from data shown in Ref. (36, 50, 63, 91); no permission is required from the copyright holder.

The Ca^2+^-mobilizing pathway is operative in gonadotrophs and is activated by GnRH as well as by pituitary adenylate cyclase-activating peptides, endothelins, ACh, vasopressin and oxytocin (50, 78–80). Among pituitary cells, a unique characteristic of mammalian gonadotrophs is the oscillatory pattern of Ca^2+^ release through IP_3_Rs. Figure 2A illustrated GnRH-induced Ca^2+^ oscillations. In contrast, αT3-1 (Figure 2B) and LβT2 gonadotrophs (not shown) release Ca^2+^ in a non-oscillatory manner when stimulated with GnRH (81, 82). GnRH-induced calcium signaling is also non-oscillatory in fish pituitary cells (83) as well as in rat Leydig cells (Figure 2B) (84). In rat gonadotrophs, the frequency of Ca^2+^ oscillations is determined by GnRH concentration and varies between 3 and 20 pulses per minute (85, 86). In neonatal rat gonadotrophs, GnRH-induced, but not IP3-stimulated, Ca^2+^ oscillations are inhibited by melatonin (87–90).

Gonadotropin-releasing hormone-induced Ca^2+^ oscillations have profound effects on electrical activity of these cells. In current-clamped gonadotrophs, GnRH-induced a transient hyperpolarization, followed by a bursting pattern episode of tall electrical spikes (Figure 2C). When the membrane was voltage-clamped, GnRH-induced current oscillations were observed (Figure 2D) (91, 92). Patterns of Ca^2+^ and current oscillations are highly comparable in the same cell and current oscillations coincide with transient hyperpolazation of PM. It is well established that Ca^2+^-activated SK channels account for coupling from the ER to PM in rat gonadotrophs (16, 38, 46, 93), whereas BK channels may also contribute to such coupling in mice gonadotrophs (40). In non-oscillatory αT3-1 gonadotrophs, GnRH stimulates L-type Ca^2+^ channels, leading to protein kinase C-dependent ERK activation (94), a process that requires dynamin GTP-ase activity (95).

The physiological relevance of bursting electrical activity in GnRH-stimulated gonadotrophs has been shown in voltage-clamped cells. By controlling the holding potential (HP) of the cell, this procedure provides a way to control the Ca^2+^ influx rate. In hyperpolarized cells with silent Ca_v_ channels, GnRH-induced current oscillations persist for about 5 min, reflecting a gradual depletion of the ER Ca^2+^ content. However, when the HP was more depolarized, many Ca_v_ channels are open and GnRH-induced current oscillations last much longer (Figure 2E), indicating that voltage-gated Ca^2+^ influx sustains signaling (91). Facilitation of Ca^2+^ influx through P2X2R channels also increases amplitudes of sustained GnRH-stimulated current oscillations (Figure 2F), a finding consistent with effect of ATP on GnRH-induced *V*_m_ oscillations and LH release (67).

The gating properties of IP_3_R channels in gonadotrophs were not studied directly, and our understanding of kinetics of opening and closing is based on analysis of GnRH/IP_3_-induced Ca^2+^/current oscillations. IP_3_ is needed to initiate the ER-dependent Ca^2+^ signaling, oscillations in intracellular IP_3_ are not required to generate oscillatory Ca^2+^ release as documented by injection of non-metabolizable IP_3_ analogs, and the concentration of IP_3_ underlines the frequency of spiking (96). Furthermore, cytosolic Ca^2+^ influences IP_3_-dependent Ca^2+^ release in these cells bidirectionally, stimulatory at lower concentrations and inhibitory at higher concentrations. The rapid stimulatory effect of Ca^2+^ on IP_3_-depenent Ca^2+^ release is shown by phase resetting of GnRH-induced oscillations by a brief pulse of voltage-gated Ca^2+^ entry (97). The inhibitory effect of high Ca^2+^ concentrations on GnRH-induced Ca^2+^ oscillations was also shown (98).

## Intercellular Signaling by Gap Junction Channels

Secretory cells are not randomly spread throughout the pituitary gland but represent very organized three-dimensional network structures critical for the proper cell-type function (54, 99). Tridimensional imaging also suggested that pituitary gonadotrophs form a homotypic network (55). These and other pituitary cells express connexin-43 (100). In general, coupling of cells through connexin gap junctions provides a pathway for the passage of ions, metabolites, small molecules, and second messengers from cell to cell, without exposure to the extracellular environment (101, 102). However, the roles of connexins in synchronization of gonadotroph activity in intact tissue have not been systematically investigated.

## Conclusion

This short review clearly indicates the complexity in expression and role of PM and ER channels in gonadotrophs. Various voltage-gated and related channels provide a background pathway for spontaneous firing of APs and Ca^2+^ signaling. In contrast to other secretory pituitary cells, spontaneous electrical activity is not coupled to exocytosis, i.e., Ca^2+^ signals generated by APs are subthreshold. However, the excitability of gonadotrophs is facilitated by activation of nicotinic AChRs, GABA_A_R, and P2X2Rs, and the accompanied Ca^2+^ signals can trigger gonadotropin secretion. Activation of GnRHR and other Ca^2+^-mobilizing receptors in gonadotrophs leads to Ca^2+^ release from ER through IP_3_R channels coupled with a rapid LH secretion, and switch in the pattern of firing of APs from tonic single spiking to periodic plateau bursting, the latter being essential for sustained Ca^2+^ signaling and LH secretion. Further studies are needed to detail the role of ion channels in intracellular signaling cascade, gene expression, Ca^2+^ secretion coupling, and mechanism of synchronous activation of gonadotrophs in intact tissue.

## Author Contributions

All the authors participated in writing; SS prepared figures.

## Conflict of Interest Statement

The authors declare that the research was conducted in the absence of any commercial or financial relationships that could be construed as a potential conflict of interest.

## References

[1] McArdle CA Roberson MS Gonadotropes and gonadotropin-releasing hormone signaling. Fourth ed In: Plant TM Zeleznik AJ, editors. Knobil and Neill’s Physiology or Reproduction. Waltham, MA: Academic Press (2015). p. 335–97.

[2] Thompson IR Kaiser UB. GnRH pulse frequency-dependent differential regulation of LH and FSH gene expression. Mol Cell Endocrinol (2014) 385:28–35. 10.1016/j.mce.2013.09.012 24056171 PMC3947649

[3] Thackray VG Mellon PL Coss D. Hormones in synergy: regulation of the pituitary gonadotropin genes. Mol Cell Endocrinol (2010) 314:192–203. 10.1016/j.mce.2009.09.003 19747958 PMC2815122

[4] Hapgood JP Sadie H van Biljon W Ronacher K. Regulation of expression of mammalian gonadotrophin-releasing hormone receptor genes. J Neuroendocrinol (2005) 17:619–38. 10.1111/j.1365-2826.2005.01353.x 16159375

[5] Kucka M Bjelobaba I Clokie SJ Klein DC Stojilkovic SS. Female-specific induction of rat pituitary dentin matrix protein-1 by GnRH. Mol Endocrinol (2013) 27:1840–55. 10.1210/me.2013-1068 24085820 PMC3805844

[6] Fowkes RC King P Burrin JM. Regulation of human glycoprotein hormone alpha-subunit gene transcription in LbetaT2 gonadotropes by protein kinase C and extracellular signal-regulated kinase 1/2. Biol Reprod (2002) 67:725–34. 10.1095/biolreprod67.3.725 12193378

[7] Kelberman D Rizzoti K Lovell-Badge R Robinson IC Dattani MT. Genetic regulation of pituitary gland development in human and mouse. Endocr Rev (2009) 30:790–829. 10.1210/er.2009-0008 19837867 PMC2806371

[8] Stojilkovic SS. Pituitary cell type-specific electrical activity, calcium signaling and secretion. Biol Res (2006) 39:403–23. 10.4067/S0716-97602006000300004 17106574

[9] Stojilkovic SS Tabak J Bertram R. Ion channels and signaling in the pituitary gland. Endocr Rev (2010) 31:845–915. 10.1210/er.2010-0005 20650859 PMC3365841

[10] Stojilkovic SS. Molecular mechanisms of pituitary endocrine cell calcium handling. Cell Calcium (2012) 51:212–21. 10.1016/j.ceca.2011.11.003 22138111 PMC3302980

[11] Millar RP Lu ZL Pawson AJ Flanagan CA Morgan K Maudsley SR. Gonadotropin-releasing hormone receptors. Endocr Rev (2004) 25:235–75. 10.1210/er.2003-0002 15082521

[12] Naor Z. Signaling by G-protein-coupled receptor (GPCR): studies on the GnRH receptor. Front Neuroendocrinol (2009) 30:10–29. 10.1016/j.yfrne.2008.07.001 18708085

[13] Yu FH Yarov-Yarovoy V Gutman GA Catterall WA Overview of molecular relationships in the voltage-gated ion channel superfamily. Pharmacol Rev (2005) 57:387–95. 10.1124/pr.57.4.13 16382097

[14] Catterall WA Goldin AL Waxman SG. International Union of Pharmacology. XLVII. Nomenclature and structure-function relationships of voltage-gated sodium channels. Pharmacol Rev (2005) 57:397–409. 10.1124/pr.57.4.5 16382098

[15] Tse A Hille B. Role of voltage-gated Na+ and Ca2+ channels in gonadotropin-releasing hormone-induced membrane potential changes in identified rat gonadotropes. Endocrinology (1993) 132:1475–81. 10.1210/endo.132.4.8384989 8384989

[16] Van Goor F Zivadinovic D Stojilkovic SS. Differential expression of ionic channels in rat anterior pituitary cells. Mol Endocrinol (2001) 15:1222–36. 10.1210/mend.15.7.0668 11435620

[17] Heyward PM Chen C Clarke IJ. Inward membrane currents and electrophysiological responses to GnRH in ovine gonadotropes. Neuroendocrinology (1995) 61:609–21. 10.1159/000126887 7544876

[18] Price CJ Goldberg JI Chang JP. Voltage-activated ionic currents in goldfish pituitary cells. Gen Comp Endocrinol (1993) 92:16–30. 10.1006/gcen.1993.1139 7505247

[19] Van Goor F Goldberg JI Chang JP. Electrical membrane properties and ionic currents in cultured goldfish gonadotrophs. Can J Physiol Pharmacol (1996) 74:729–43. 10.1139/y96-067 8909786

[20] Waring DW Turgeon JL. Estradiol inhibition of voltage-activated and gonadotropin-releasing hormone-induced currents in mouse gonadotrophs. Endocrinology (2006) 147:5798–805. 10.1210/en.2006-1112 16946005

[21] Wen S Schwarz JR Niculescu D Dinu C Bauer CK Hirdes W Functional characterization of genetically labeled gonadotropes. Endocrinology (2008) 149:2701–11. 10.1210/en.2007-1502 18325995

[22] Bosma MM Hille B. Electrophysiological properties of a cell line of the gonadotrope lineage. Endocrinology (1992) 130:3411–20. 10.1210/endo.130.6.1317783 1317783

[23] Tiwari JK Sikdar SK. Voltage gated Na+ channels contribute to membrane voltage fluctuation in alphaT3-1 pituitary gonadotroph cells. Neurosci Lett (1998) 242:167–71. 10.1016/S0304-3940(98)00046-9 9530932

[24] Kucka M Kretschmannova K Murano T Wu CP Zemkova H Ambudkar SV Dependence of multidrug resistance protein-mediated cyclic nucleotide efflux on the background sodium conductance. Mol Pharmacol (2010) 77:270–9. 10.1124/mol.109.059386 19903828 PMC2812068

[25] Sankaranarayanan S Simasko SM. A role for a background sodium current in spontaneous action potentials and secretion from rat lactotrophs. Am J Physiol (1996) 271:C1927–34. 8997194 10.1152/ajpcell.1996.271.6.C1927

[26] Catterall WA Perez-Reyes E Snutch TP Striessnig J. International Union of Pharmacology. XLVIII. Nomenclature and structure-function relationships of voltage-gated calcium channels. Pharmacol Rev (2005) 57:411–25. 10.1124/pr.57.4.5 16382099

[27] Mason WT Sikdar SK. Characteristics of voltage-gated Ca2+ currents in ovine gonadotrophs. J Physiol (1989) 415:367–91. 10.1113/jphysiol.1989.sp017726 2561790 PMC1189181

[28] Gutman GA Chandy KG Grissmer S Lazdunski M McKinnon D Pardo LA International Union of Pharmacology. LIII. Nomenclature and molecular relationships of voltage-gated potassium channels. Pharmacol Rev (2005) 57:473–508. 10.1124/pr.57.4.10 16382104

[29] Cowley MA Chen C Clarke IJ. Estrogen transiently increases delayed rectifier, voltage-dependent potassium currents in ovine gonadotropes. Neuroendocrinology (1999) 69:254–60. 10.1159/000054426 10207277

[30] Haug TM Hodne K Weltzien FA Sand O. Electrophysiological properties of pituitary cells in primary culture from Atlantic cod (*Gadus morhua*). Neuroendocrinology (2007) 86:38–47. 10.1159/000103867 17565196

[31] Mei YA Soriani O Castel H Vaudry H Cazin L. Adenosine potentiates the delayed-rectifier potassium conductance but has no effect on the hyperpolarization-activated Ih current in frog melanotrophs. Brain Res (1998) 793:271–8. 10.1016/S0006-8993(98)00184-X 9630670

[32] Herrington J Lingle CJ. Multiple components of voltage-dependent potassium current in normal rat anterior pituitary cells. J Neurophysiol (1994) 72:719–29. 7983530 10.1152/jn.1994.72.2.719

[33] Chen C Zhang J Vincent JD Israel JM. Somatostatin increases voltage-dependent potassium currents in rat somatotrophs. Am J Physiol (1990) 259:C854–61. 1979715 10.1152/ajpcell.1990.259.6.C854

[34] Hirdes W Dinu C Bauer CK Boehm U Schwarz JR Gonadotropin-releasing hormone inhibits ether-a-go-go-related gene K+ currents in mouse gonadotropes. Endocrinology (2010) 151:1079–88. 10.1210/en.2009-0718 20068004

[35] Wei AD Gutman GA Aldrich R Chandy KG Grissmer S Wulff H International Union of Pharmacology. LII. Nomenclature and molecular relationships of calcium-activated potassium channels. Pharmacol Rev (2005) 57:463–72. 10.1124/pr.57.4.9 16382103

[36] Stojilkovic SS Zemkova H Van Goor F. Biophysical basis of pituitary cell type-specific Ca2+ signaling-secretion coupling. Trends Endocrinol Metab (2005) 16:152–9. 10.1016/j.tem.2005.03.003 15860411

[37] Hodne K Strandabo RA von Krogh K Nourizadeh-Lillabadi R Sand O Weltzien FA Electrophysiological differences between fshb- and lhb-expressing gonadotropes in primary culture. Endocrinology (2013) 154:3319–30. 10.1210/en.2013-1164 23836032

[38] Kukuljan M Stojilkovic SS Rojas E Catt KJ. Apamin-sensitive potassium channels mediate agonist-induced oscillations of membrane potential in pituitary gonadotrophs. FEBS Lett (1992) 301:19–22. 10.1016/0014-5793(92)80201-Q 1333410

[39] Tse A Hille B. GnRH-induced Ca2+ oscillations and rhythmic hyperpolarizations of pituitary gonadotropes. Science (1992) 255:462–4. 10.1126/science.1734523 1734523

[40] Waring DW Turgeon JL. Ca2+-activated K+ channels in gonadotropin-releasing hormone-stimulated mouse gonadotrophs. Endocrinology (2009) 150:2264–72. 10.1210/en.2008-1442 19106218 PMC2671892

[41] Kretschmannova K Kucka M Gonzalez-Iglesias AE Stojilkovic SS. The expression and role of hyperpolarization-activated and cyclic nucleotide-gated channels in endocrine anterior pituitary cells. Mol Endocrinol (2012) 26:153–64. 10.1210/me.2011-1207 22135067 PMC3248322

[42] Craven KB Zagotta WN. CNG and HCN channels: two peas, one pod. Annu Rev Physiol (2006) 68:375–401. 10.1146/annurev.physiol.68.040104.134728 16460277

[43] Tomic M Kucka M Kretschmannova K Li S Nesterova M Stratakis CA Role of nonselective cation channels in spontaneous and protein kinase A-stimulated calcium signaling in pituitary cells. Am J Physiol Endocrinol Metab (2011) 301:E370–9. 10.1152/ajpendo.00130.2011 21586701 PMC3154538

[44] Clapham DE Julius D Montell C Schultz G International Union of Pharmacology. XLIX. Nomenclature and structure-function relationships of transient receptor potential channels. Pharmacol Rev (2005) 57:427–50. 10.1124/pr.57.4.6 16382100

[45] Beck A Gotz V Qiao S Weissgerber P Flockerzi V Freichel M Functional characterization of transient receptor potential (TRP) channel C5 in female murine gonadotropes. Endocrinology (2017) 158:887–902. 10.1210/en.2016-1810 28324107

[46] Vergara L Rojas E Stojilkovic SS. A novel calcium-activated apamin-insensitive potassium current in pituitary gonadotrophs. Endocrinology (1997) 138:2658–64. 10.1210/endo.138.7.5220 9202201

[47] Van Goor F Li YX Stojilkovic SS. Paradoxical role of large-conductance calcium-activated K+ (BK) channels in controlling action potential-driven Ca2+ entry in anterior pituitary cells. J Neurosci (2001) 21:5902–15. 11487613 10.1523/JNEUROSCI.21-16-05902.2001 PMC6763171

[48] Tsaneva-Atanasova K Sherman A van Goor F Stojilkovic SS. Mechanism of spontaneous and receptor-controlled electrical activity in pituitary somatotrophs: experiments and theory. J Neurophysiol (2007) 98:131–44. 10.1152/jn.00872.2006 17493919

[49] Chen C Zhang J Vincent JD Israel JM. Sodium and calcium currents in action potentials of rat somatotrophs: their possible functions in growth hormone secretion. Life Sci (1990) 46:983–9. 10.1016/0024-3205(90)90021-I 2157930

[50] Zemkova H Kucka M Bjelobaba I Tomic M Stojilkovic SS. Multiple cholinergic signaling pathways in pituitary gonadotrophs. Endocrinology (2013) 154:421–33. 10.1210/en.2012-1554 23161872 PMC3529387

[51] Stojilkovic SS Kretschmannova K Tomic M Stratakis CA. Dependence of the excitability of pituitary cells on cyclic nucleotides. J Neuroendocrinol (2012) 24:1183–200. 10.1111/j.1365-2826.2012.02335.x 22564128 PMC3421050

[52] Van Goor F Zivadinovic D Martinez-Fuentes AJ Stojilkovic SS. Dependence of pituitary hormone secretion on the pattern of spontaneous voltage-gated calcium influx. Cell type-specific action potential secretion coupling. J Biol Chem (2001) 276:33840–6. 10.1074/jbc.M105386200 11457854

[53] Schlegel W Winiger BP Mollard P Vacher P Wuarin F Zahnd GR Oscillations of cytosolic Ca2+ in pituitary cells due to action potentials. Nature (1987) 329:719–21. 10.1038/329719a0 2444888

[54] Bonnefont X Lacampagne A Sanchez-Hormigo A Fino E Creff A Mathieu MN Revealing the large-scale network organization of growth hormone-secreting cells. Proc Natl Acad Sci U S A (2005) 102:16880–5. 10.1073/pnas.0508202102 16272219 PMC1277257

[55] Budry L Lafont C El Yandouzi T Chauvet N Conejero G Drouin J Related pituitary cell lineages develop into interdigitated 3D cell networks. Proc Natl Acad Sci U S A (2011) 108:12515–20. 10.1073/pnas.1105929108 21746936 PMC3145718

[56] Chauvet N El-Yandouzi T Mathieu MN Schlernitzauer A Galibert E Lafont C Characterization of adherens junction protein expression and localization in pituitary cell networks. J Endocrinol (2009) 202:375–87. 10.1677/JOE-09-0153 19505949

[57] Connolly CN Wafford KA. The Cys-loop superfamily of ligand-gated ion channels: the impact of receptor structure on function. Biochem Soc Trans (2004) 32:529–34. 10.1042/bst0320529 15157178

[58] Mayer ML Olson R Gouaux E. Mechanisms for ligand binding to GluR0 ion channels: crystal structures of the glutamate and serine complexes and a closed apo state. J Mol Biol (2001) 311:815–36. 10.1006/jmbi.2001.4884 11518533

[59] Coddou C Yan Z Obsil T Huidobro-Toro JP Stojilkovic SS. Activation and regulation of purinergic P2X receptor channels. Pharmacol Rev (2011) 63:641–83. 10.1124/pr.110.003129 21737531 PMC3141880

[60] Owens DF Kriegstein AR. Is there more to GABA than synaptic inhibition? Nat Rev Neurosci (2002) 3:715–27. 10.1038/nrn919 12209120

[61] Mijiddorj T Kanasaki H Sukhbaatar U Oride A Kyo S. DS1, a delta subunit-containing GABA(A) receptor agonist, increases gonadotropin subunit gene expression in mouse pituitary gonadotrophs. Biol Reprod (2015) 92:45. 10.1095/biolreprod.114.123893 25519184

[62] Virmani MA Stojilkovic SS Catt KJ. Stimulation of luteinizing hormone release by gamma-aminobutyric acid (GABA) agonists: mediation by GABAA-type receptors and activation of chloride and voltage-sensitive calcium channels. Endocrinology (1990) 126:2499–505. 10.1210/endo-126-5-2499 2158428

[63] Zemkova HW Bjelobaba I Tomic M Zemkova H Stojilkovic SS. Molecular, pharmacological and functional properties of GABA(A) receptors in anterior pituitary cells. J Physiol (2008) 586:3097–111. 10.1113/jphysiol.2008.153148 18450776 PMC2538769

[64] Hogg RC Raggenbass M Bertrand D. Nicotinic acetylcholine receptors: from structure to brain function. Rev Physiol Biochem Pharmacol (2003) 147:1–46. 10.1007/s10254-003-0005-1 12783266

[65] Zouridakis M Zisimopoulou P Poulas K Tzartos SJ. Recent advances in understanding the structure of nicotinic acetylcholine receptors. IUBMB Life (2009) 61:407–23. 10.1002/iub.170 19319967

[66] Tomic M Jobin RM Vergara LA Stojilkovic SS. Expression of purinergic receptor channels and their role in calcium signaling and hormone release in pituitary gonadotrophs. Integration of P2 channels in plasma membrane- and endoplasmic reticulum-derived calcium oscillations. J Biol Chem (1996) 271:21200–8. 10.1074/jbc.271.35.21200 8702891

[67] Zemkova H Balik A Jiang Y Kretschmannova K Stojilkovic SS. Roles of purinergic P2X receptors as pacemaking channels and modulators of calcium-mobilizing pathway in pituitary gonadotrophs. Mol Endocrinol (2006) 20:1423–36. 10.1210/me.2005-0508 16543406

[68] Koshimizu T Tomic M Van Goor F Stojilkovic SS. Functional role of alternative splicing in pituitary P2X2 receptor-channel activation and desensitization. Mol Endocrinol (1998) 12:901–13. 10.1210/mend.12.7.0129 9658396

[69] He ML Gonzalez-Iglesias AE Tomic M Stojilkovic SS. Release and extracellular metabolism of ATP by ecto-nucleotidase eNTPDase 1-3 in hypothalamic and pituitary cells. Purinergic Signal (2005) 1:135–44. 10.1007/s11302-005-6208-y 18404498 PMC2096527

[70] Allen-Worthington K Xie J Brown JL Edmunson AM Dowling A Navratil AM The F0F1 ATP synthase complex localizes to membrane rafts in gonadotrope cells. Mol Endocrinol (2016) 30:996–1011. 10.1210/me.2015-1324 27482602 PMC5414608

[71] Li S Bjelobaba I Stojilkovic SS. Interactions of Pannexin1 channels with purinergic and NMDA receptor channels. Biochim Biophys Acta (2017). 10.1016/j.bbamem.2017.03.025 28389204 PMC5628093

[72] Li S Bjelobaba I Yan Z Kucka M Tomic M Stojilkovic SS. Expression and roles of pannexins in ATP release in the pituitary gland. Endocrinology (2011) 152:2342–52. 10.1210/en.2010-1216 21467198 PMC3100624

[73] Zemkova H Kucka M Li S Gonzalez-Iglesias AE Tomic M Stojilkovic SS. Characterization of purinergic P2X4 receptor channels expressed in anterior pituitary cells. Am J Physiol Endocrinol Metab (2010) 298:E644–51. 10.1152/ajpendo.00558.2009 20009029 PMC2838522

[74] He ML Gonzalez-Iglesias AE Stojilkovic SS. Role of nucleotide P2 receptors in calcium signaling and prolactin release in pituitary lactotrophs. J Biol Chem (2003) 278:46270–7. 10.1074/jbc.M309005200 12970352

[75] Stojilkovic SS. Purinergic regulation of hypothalamopituitary functions. Trends Endocrinol Metab (2009) 20:460–8. 10.1016/j.tem.2009.05.005 19800813 PMC2766266

[76] Li S Tomic M Stojilkovic SS. Characterization of novel Pannexin 1 isoforms from rat pituitary cells and their association with ATP-gated P2X channels. Gen Comp Endocrinol (2011) 174:202–10. 10.1016/j.ygcen.2011.08.019 21907716 PMC3195874

[77] Putney JW. Capacitative calcium entry: from concept to molecules. Immunol Rev (2009) 231:10–22. 10.1111/j.1600-065X.2009.00810.x 19754887

[78] Rawlings SR Demaurex N Schlegel W. Pituitary adenylate cyclase-activating polypeptide increases [Ca2]i in rat gonadotrophs through an inositol trisphosphate-dependent mechanism. J Biol Chem (1994) 269:5680–6. 7907085

[79] Stojilkovic SS Merelli F Iida T Krsmanovic LZ Catt KJ. Endothelin stimulation of cytosolic calcium and gonadotropin secretion in anterior pituitary cells. Science (1990) 248:1663–6. 10.1126/science.2163546 2163546

[80] Evans JJ Forrest-Owen W McArdle CA. Oxytocin receptor-mediated activation of phosphoinositidase C and elevation of cytosolic calcium in the gonadotrope-derived alphaT3-1 cell line. Endocrinology (1997) 138:2049–55. 10.1210/endo.138.5.5138 9112404

[81] Thomas P Mellon PL Turgeon J Waring DW. The L beta T2 clonal gonadotrope: a model for single cell studies of endocrine cell secretion. Endocrinology (1996) 137:2979–89. 10.1210/endo.137.7.8770922 8770922

[82] Naidich M Shterntal B Furman R Pawson AJ Jabbour HN Morgan K Elucidation of mechanisms of the reciprocal cross talk between gonadotropin-releasing hormone and prostaglandin receptors. Endocrinology (2010) 151:2700–12. 10.1210/en.2009-1335 20392830

[83] Strandabo RA Hodne K Ager-Wick E Sand O Weltzien FA Haug TM. Signal transduction involved in GnRH2-stimulation of identified LH-producing gonadotropes from lhb-GFP transgenic medaka (*Oryzias latipes*). Mol Cell Endocrinol (2013) 372:128–39. 10.1016/j.mce.2013.03.022 23562421

[84] Tomic M Dufau ML Catt KJ Stojilkovic SS. Calcium signaling in single rat Leydig cells. Endocrinology (1995) 136:3422–9. 10.1210/endo.136.8.7628378 7628378

[85] Tomic M Cesnjaj M Catt KJ Stojilkovic SS. Developmental and physiological aspects of Ca2+ signaling in agonist-stimulated pituitary gonadotrophs. Endocrinology (1994) 135:1762–71. 10.1210/endo.135.5.7956899 7956899

[86] Stojilkovic SS Torsello A Iida T Rojas E Catt KJ. Calcium signaling and secretory responses in agonist-stimulated pituitary gonadotrophs. J Steroid Biochem Mol Biol (1992) 41:453–67. 10.1016/0960-0760(92)90371-O 1373299

[87] Vanecek J Klein DC. Sodium-dependent effects of melatonin on membrane potential of neonatal rat pituitary cells. Endocrinology (1992) 131:939–46. 10.1210/endo.131.2.1322288 1322288

[88] Vanecek J Klein DC. Melatonin inhibits gonadotropin-releasing hormone-induced elevation of intracellular Ca2+ in neonatal rat pituitary cells. Endocrinology (1992) 130:701–7. 10.1210/en.130.2.701 1733718

[89] Zemkova H Vanecek J. Inhibitory effect of melatonin on gonadotropin-releasing hormone-induced Ca2+ oscillations in pituitary cells of newborn rats. Neuroendocrinology (1997) 65:276–83. 10.1159/000127185 9142999

[90] Zemkova H Vanecek J. Differences in gonadotropin-releasing hormone-induced calcium signaling between melatonin-sensitive and melatonin-insensitive neonatal rat gonadotrophs. Endocrinology (2000) 141:1017–26. 10.1210/endo.141.3.7351 10698178

[91] Kukuljan M Vergara L Stojilkovic SS. Modulation of the kinetics of inositol 1,4,5-trisphosphate-induced [Ca^2+^]i oscillations by calcium entry in pituitary gonadotrophs. Biophys J (1997) 72:698–707. 10.1016/S0006-3495(97)78706-X 9017197 PMC1185595

[92] Kukuljan M Rojas E Catt KJ Stojilkovic SS. Membrane potential regulates inositol 1,4,5-trisphosphate-controlled cytoplasmic Ca2+ oscillations in pituitary gonadotrophs. J Biol Chem (1994) 269:4860–5. 8106457

[93] Tse A Lee AK. Arginine vasopressin triggers intracellular calcium release, a calcium-activated potassium current and exocytosis in identified rat corticotropes. Endocrinology (1998) 139:2246–52. 10.1210/endo.139.5.5999 9564830

[94] Dang AK Murtazina DA Magee C Navratil AM Clay CM Amberg GC. GnRH evokes localized subplasmalemmal calcium signaling in gonadotropes. Mol Endocrinol (2014) 28:2049–59. 10.1210/me.2014-1208 25333516 PMC4250365

[95] Edwards BS Dang AK Murtazina DA Dozier MG Whitesell JD Khan SA Dynamin is required for GnRH signaling to L-type calcium channels and activation of ERK. Endocrinology (2016) 157:831–43. 10.1210/en.2015-1575 26696122 PMC4733113

[96] Stojilkovic SS Kukuljan M Tomic M Rojas E Catt KJ. Mechanism of agonist-induced [Ca^2+^]i oscillations in pituitary gonadotrophs. J Biol Chem (1993) 268:7713–20. 8463300

[97] Vergara LA Stojilkovic SS Rojas E. GnRH-induced cytosolic calcium oscillations in pituitary gonadotrophs: phase resetting by membrane depolarization. Biophys J (1995) 69:1606–14. 10.1016/S0006-3495(95)80033-0 8534831 PMC1236391

[98] Stojilkovic SS Tomic M Kukuljan M Catt KJ. Control of calcium spiking frequency in pituitary gonadotrophs by a single-pool cytoplasmic oscillator. Mol Pharmacol (1994) 45:1013–21. 8190091

[99] Fauquier T Guerineau NC McKinney RA Bauer K Mollard P. Folliculostellate cell network: a route for long-distance communication in the anterior pituitary. Proc Natl Acad Sci U S A (2001) 98:8891–6. 10.1073/pnas.151339598 11438713 PMC37531

[100] Yamamoto T Hossain MZ Hertzberg EL Uemura H Murphy LJ Nagy JI. Connexin43 in rat pituitary: localization at pituicyte and stellate cell gap junctions and within gonadotrophs. Histochemistry (1993) 100:53–64. 10.1007/BF00268878 8226109

[101] Harris AL. Connexin channel permeability to cytoplasmic molecules. Prog Biophys Mol Biol (2007) 94:120–43. 10.1016/j.pbiomolbio.2007.03.011 17470375 PMC1995164

[102] Cruciani V Mikalsen SO. The vertebrate connexin family. Cell Mol Life Sci (2006) 63:1125–40. 10.1007/s00018-005-5571-8 16568237 PMC11136263

